# DGK Positionspapier – Qualitätssicherung zur Durchführung der Katheterablation von Vorhofflimmern: *Executive Summary*

**DOI:** 10.1007/s00399-026-01149-2

**Published:** 2026-05-19

**Authors:** Daniel Steven, Julian Chun, Isabel Deisenhofer, Thomas Deneke, Maria Papathanasiou, Boris Schmidt, Andreas Rillig, Stephan Willems, Maura M. Zylla, Christian Veltmann, Lars Eckardt

**Affiliations:** 1 Abteilung für Elektrophysiologie, Herzzentrum Uniklinik Köln, Kerpener Str. 62, 50937 Köln, Deutschland; 2 Medizinische Klinik III – CCB, Agaplesion Markus Krankenhaus, Frankfurt am Main, Deutschland; 3 Abteilung für Elektrophysiologie, Deutsches Herzzentrum München, München, Deutschland; 4 Klinik für Kardiologie und Rhythmologie, Klinikum Nürnberg Süd, Nürnberg, Deutschland; 5 Med. Klinik III – Kardiologie, Angiologie, Universitätsklinikum Frankfurt, Frankfurt am Main, Deutschland; 6 Klinik für Kardiologie mit Schwerpunkt Elektrophysiologie, Universitäres Herz- und Gefäßzentrum Hamburg, Hamburg, Deutschland; 7 Kardiologie & internistische Intensivmedizin, Asklepios Klinik St. Georg, Hamburg, Deutschland; 8 Klinik für Innere Med. III, Kardiologie, Angiologie u. Pneumologie, Universitätsklinikum Heidelberg, Heidelberg, Deutschland; 9 Elektrophysiologie Bremen im Klinikum Links der Weser, Bremen, Deutschland; 10 Klinik für Kardiologie II – Rhythmologie, Universitätsklinikum Münster, Münster, Deutschland

**Keywords:** Arrhythmie, Vorhofflimmern, Antikoagulation, Katheterablation, Herzinsuffizienz, Arrhythmia, Atrial fibrillation, Anticoagulation, Catheter ablation, Heart failure

## Abstract

Das aktualisierte Positionspapier der DGK zur Katheterablation von Vorhofflimmern (AF) [1] stellt die aktuelle Evidenz, Techniken und Qualitätsstandards dar, die sich seit 2017 gemeinsam mit Indikation, Technik und Rolle der Ablation in der Therapie deutlich gewandelt haben. Die Pulmonalvenenisolation (PVI) bleibt zentraler Bestandteil der AF-Ablation. Neben etablierten Verfahren wie Radiofrequenz- und Kryoablation gewinnt die Pulsed-Field-Ablation (PFA) an Bedeutung. Bei persistierendem AF fehlen klare Empfehlungen über die PVI hinaus, trotz zunehmender Daten für ergänzende Ablationsstrategien. Die Versorgungssituation zeigt eine starke Zunahme der Ablationen, wobei die tagesgleiche Entlassung nur für selektierte Patienten empfohlen wird. Die präzise Patientenselektion, inklusive Anamnese, Risikofaktoren und Bildgebung sind entscheidend für den Erfolg und die Sicherheit. Die Bedeutung von periprozeduralem Management und strukturierter Nachsorge wird hervorgehoben. Komplikationen wie Perikardtamponade, Schlaganfall und Phrenikusparese erfordern strukturierte Abläufe und erfahrene Teams. Besonders bei Patienten mit Herzinsuffizienz zeigt sich die Ablation als potenziell prognoseverbessernd. Die Zertifizierung von Zentren durch die DGK dient der Qualitätssicherung. Neue Entwicklungen wie KI-gestützte Ablationsplanung und Studien zur OAK-Strategie nach Ablation werden die Praxis weiter verändern.

## Abstract (en)

The updated German Society of Cardiology (DGK) position paper on catheter ablation of atrial fibrillation (AF) [1] presents the current evidence, techniques, and quality standards, which have evolved significantly since 2017 alongside indications, technology, and the role of ablation in therapy. Pulmonary vein isolation (PVI) remains the cornerstone of AF ablation. In addition to established procedures such as radiofrequency and cryoballoon ablation, pulsed field ablation (PFA) is gaining importance. For persistent AF, clear recommendations beyond PVI are still lacking, despite growing evidence supporting adjunctive ablation strategies. The healthcare landscape shows a marked increase in ablations, with same day discharge procedures recommended only for carefully selected patients. Precise patient selection—including medical history, risk factors, and imaging—is critical for procedural success and safety. The importance of periprocedural management and structured follow-up is emphasized. Complications such as pericardial tamponade, stroke, and phrenic nerve injury require structured protocols and experienced teams. Particularly in patients with heart failure, ablation may offer prognostic benefits. Certification of centers by the DGK supports quality assurance. New developments, such as artificial intelligence (AI)-assisted ablation planning and studies on postablation anticoagulation strategies, are expected to further influence clinical practice.

Ein Positionspapier der Deutschen Gesellschaft für Kardiologie (DGK) zu den Qualitätskriterien der Katheterablation von Vorhofflimmern (AF) wurde erstmals 2017 publiziert und liegt seit Ende des vergangenen Jahres in aktualisierter Fassung vor. Die Überarbeitung war erforderlich, da sich in den letzten Jahren wesentliche Veränderungen bei Indikation, technologischen Voraussetzungen und praktischer Durchführung der Katheterablation von AF ergeben haben. So haben sich sowohl das Verständnis der Rhythmuskontrolle als auch die Rolle der Ablation als bedeutsame First-Line-Therapie weiterentwickelt. Dies spiegelt sich auch in der 2025 erstmals veröffentlichten S3-Leitlinie zur Behandlung des AF wider, in der die Rhythmuskontrolle in den Vordergrund gerückt ist [2]. Zudem haben Ablationsstrategien insbesondere bei Herzinsuffizienz heute einen deutlich höheren Stellenwert als noch 2017.

Die Arbeitsgruppe Elektrophysiologie (AGEP) der DGK hat sich in den vergangenen Jahren intensiv mit Fragen der Qualitätssicherung beschäftigt, da diesem Thema künftig eine noch größere Bedeutung zukommen dürfte. Im Jahr 2022 stellte die AGEP beim Gemeinsamen Bundesausschuss (G-BA) einen Antrag auf Einführung einer strukturierten Qualitätssicherung in der invasiven Elektrophysiologie. Dieser wurde mit der Begründung abgelehnt, dass bereits etablierte Qualitätsprüfungen bestünden und eine zusätzliche Struktur damals nicht vorgesehen sei. Unabhängig davon hat die DGK bereits 2012 ein Curriculum für Elektrophysiologie [3] entwickelt, das aktuell überarbeitet wird. Es bildet die Grundlage für die Zertifizierung von Einzelpersonen und Einrichtungen und berücksichtigt personelle und technische Ausstattung, Infrastruktur sowie Leistungszahlen. Eine spezifische Zertifizierung für Vorhofflimmerzentren besteht seit 2020. Im April 2026 sind deutschlandweit 107 Zentren zertifiziert, davon 57 bereits rezertifiziert.

Das Positionspapier zu Qualitätskriterien der Vorhofflimmerablation versteht sich als praxisorientierter Leitfaden für Qualitätsstandards, mit dem Ziel, die Versorgung von Patientinnen und Patienten vor, während und nach einer Katheterablation bei Vorhofflimmern zu optimieren.

## Versorgungssituation und tagesgleiche Entlassung

Mit der steigenden AF-Prävalenz wächst der Bedarf an effektiven Therapiestrategien zur Symptomkontrolle und zur Reduktion AF-assoziierter Komplikationen. Entsprechend ist in den letzten Jahren sowohl die Zahl der in Deutschland durchgeführten Katheterablationen als auch die Zahl der Zentren mit interventionellem Angebot deutlich gestiegen. Dennoch wird nur ein vergleichsweise kleiner Teil der betroffenen Personen tatsächlich abladiert [4, 5].

Im Jahr 2024 wurden in Deutschland mehr als 138.000 Katheterablationen durchgeführt, überwiegend zur Behandlung von AF [5]. Gleichzeitig zeigt sich eine erhebliche Heterogenität zwischen den Zentren: Mehr als ein Drittel der Einrichtungen führte 2024 weniger als 150 Ablationen pro Jahr durch. Der Anstieg der Prozedurzahlen unterstreicht den wachsenden Bedarf an interventioneller AF-Therapie, macht aber zugleich deutlich, wie wichtig klar definierte intra- und periprozedurale Qualitätsstandards für Effektivität und Sicherheit sind.

Der Großteil der Ablationen erfolgt aktuell stationär. Angesichts steigender Fallzahlen und begrenzter personeller sowie struktureller Ressourcen wird zunehmend die Möglichkeit einer tagesgleichen Entlassung („same day discharge“) diskutiert. Mit der Einführung der Hybrid-DRG im Januar 2026 ist nunmehr die Vorhofflimmerablation ohne stationäre Übernachtung abrechenbar. Metaanalysen mit mehr als 150.000 Fällen zeigen, dass bei sorgfältig selektionierten Patienten keine erhöhte Komplikationsrate gegenüber einer stationären Überwachung über Nacht besteht [6]. Allerdings beruht ein großer Teil dieser Evidenz auf retrospektiven Analysen; randomisiert-kontrollierte Studien liegen bislang nur in begrenztem Umfang vor. Hinzu kommen strukturelle Herausforderungen, insbesondere die Notwendigkeit einer engmaschigen und qualitätsgesicherten Nachsorge. Vor diesem Hintergrund empfiehlt die DGK eine tagesgleiche Entlassung nur für ein streng selektioniertes Patientenkollektiv und unter klar definierten Anforderungen an Prozedur und Nachsorge [7]. Dabei müssen dieselben Qualitätsmaßstäbe gelten wie in zertifizierten Vorhofflimmerzentren bei stationärer Versorgung. Da eine strukturierte Qualitätssicherung für „same day discharge“ AF-Ablationen derzeit nicht etabliert ist, empfiehlt die DGK eine überwiegend stationäre Durchführung.

### Fazit

Der steigende Bedarf an Katheterablationen bei Vorhofflimmern spiegelt die wachsende Evidenz, die zunehmenden Prozedurzahlen und die kontinuierliche Zunahme abladierender Zentren wider. Aufgrund der begrenzten Datenlage zur „same-day discharge“ AF-Ablation, der prozeduralen Komplexität und der unterschiedlichen Risikoprofile der Patienten wird für die Mehrzahl der Eingriffe aktuell eine stationäre Durchführung empfohlen.

## Periprozedurales Management und Risikofaktoren

Das periprozedurale Management ist ein zentraler Baustein für Erfolg und Sicherheit der Katheterablation. Bereits präprozedural ist eine sorgfältige Aufarbeitung der individuellen Anamnese erforderlich. Dabei sollte insbesondere zwischen paroxysmalem, eher triggerinduziertem AF und persistierenden Formen unterschieden werden, da sich hieraus unterschiedliche Erfolgsaussichten ergeben.

Besondere Bedeutung hat zudem der Zeitpunkt der Intervention im Krankheitsverlauf. In den vergangenen Jahren hat sich gezeigt, dass eine frühe Ablation mit besseren Ergebnissen verbunden ist. Länger bestehendes AF führt zu strukturellen Veränderungen im Sinne eines atrialen Remodelings mit zunehmender Fibrosierung, was die Erfolgswahrscheinlichkeit der Ablation vermindern kann. Als praktikabler Parameter hat sich die „diagnosis-to-ablation time“ etabliert. Studien belegen, dass insbesondere eine Ablation innerhalb des ersten Jahres nach Diagnose mit einer höheren Arrhythmiefreiheit einhergeht.

Neben dem Zeitpunkt spielen die systematische Diagnose und Behandlung von Risikofaktoren eine wesentliche Rolle. Zu den wichtigsten Einflussgrößen zählen arterielle Hypertonie, Herzinsuffizienz, Diabetes mellitus, Adipositas, obstruktive Schlafapnoe und Alkoholkonsum. In den letzten Jahren konnte überzeugend gezeigt werden, dass eine konsequente Optimierung dieser Faktoren den mittel- und langfristigen Erfolg der Katheterablation wesentlich beeinflusst. Dazu gehören eine leitliniengerechte Therapie der Herzinsuffizienz, eine gute Blutdruck- und Blutzuckereinstellung sowie die Behandlung einer Schlafapnoe. Ergänzend kommt dem Lebensstilmanagement eine wichtige Rolle zu, vor allem durch Gewichtsreduktion, regelmäßige körperliche Aktivität und Alkoholkarenz.

Auch das Ausmaß des atrialen Remodelings, das bis zu einer atrialen Kardiomyopathie reichen kann, beeinflusst den Ablationserfolg. Ein fortgeschrittenes Remodeling ist nicht nur mit einer geringeren Erfolgswahrscheinlichkeit assoziiert, sondern erhöht auch das Risiko intraatrialer Thromben und kann die Wahl der Ablationsstrategie beeinflussen, etwa durch zusätzliche Mappingverfahren oder substratbasierte Konzepte.

Die präprozedurale Bildgebung dient der genauen Beurteilung der kardialen Anatomie und ist für die Planung bedeutsam. Neben der obligaten transthorakalen Echokardiographie können je nach Fragestellung CT oder MRT eingesetzt werden. Die CT bietet eine hohe räumliche Auflösung und erlaubt zugleich einen Thrombenausschluss; die MRT ermöglicht eine strahlenfreie Gewebecharakterisierung, ist jedoch aufgrund von Verfügbarkeit und/oder technischen Limitationen nicht in allen Fällen gleichwertig einsetzbar.

Ein zentraler Bestandteil des periprozeduralen Managements ist der Ausschluss intrakardialer Thromben, da thromboembolische Ereignisse zu den schwerwiegendsten Komplikationen gehören. Die Indikation zur präprozeduralen Bildgebung sollte risikoadaptiert erfolgen. Während das Risiko bei paroxysmalem AF und niedrigem CHA_2_DS_2_-VA-Score (z. B. ≤ 2 Punkte) gering ist, steigt es bei persistierendem Vorhofflimmern, höherem Risikoscore und bestimmten strukturellen Herzerkrankungen deutlich an. In diesen Fällen sollte ein Thrombenausschluss auch unter laufender Antikoagulation großzügig erfolgen. Eine aktuelle Bildgebung ist zudem besonders dann angezeigt, wenn thromboembolische Ereignisse oder Vorhofthromben bekannt sind, relevante strukturelle Herzerkrankungen (z. B. Amyloidose, hypertrophe Kardiomyopathie) vorliegen oder eine insuffiziente Antikoagulation besteht [8].

Die Antikoagulation ist ein weiterer zentraler Pfeiler des periprozeduralen Managements. Sie orientiert sich am individuellen thromboembolischen Risiko und wird in der Regel ab einem CHA_2_DS_2_-VA-Score von ≥ 2 empfohlen. Ab einem CHA_2_DS_2_-VA-Score von 1 sollte sie erwogen werden. Unabhängig davon sollte vor einer Katheterablation bei allen Patienten eine mindestens dreiwöchige effektive orale Antikoagulation erfolgen. Aktuelle internationale Leitlinien favorisieren direkte orale Antikoagulanzien (DOAK) gegenüber Vitamin-K-Antagonisten. Die Ablation selbst sollte unter kontinuierlicher Antikoagulation durchgeführt werden; bei Vitamin-K-Antagonisten wird ein therapeutischer INR angestrebt, bei DOAK sollte in der Regel höchstens eine Dosis ausgelassen werden.

Zusammenfassend ist die sorgfältige Analyse der Anamnese entscheidend, um Prognose und Erfolgsaussichten einer Katheterablation realistisch einschätzen zu können. Zugleich bleibt die konsequente Vermeidung von Komplikationen, insbesondere thromboembolischer Ereignisse, ein zentrales Ziel, das eine strukturierte und individuell angepasste Antikoagulation vor, während und nach der Ablation erfordert.

## Aktuelle Datenlage und Indikationsstellung

Auf Grundlage der aktuellen Evidenz und der kontinuierlichen Weiterentwicklung der Ablationstechnologien sind die Leitlinienempfehlungen der European Society of Cardiology (ESC) andere internationale Empfehlungen und die nationale AWMF S3-Leitlinie zur Katheterablation in den letzten Jahren gestärkt und weiter differenziert worden [2, 9]. Primäres Ziel der Katheterablation bleibt die Reduktion der Arrhythmielast und der damit verbundenen Symptome. Darüber hinaus kann eine erfolgreiche Ablation die Progression der Erkrankung verlangsamen und das Fortschreiten des atrialen Remodelings günstig beeinflussen.

Ein prognostischer Nutzen der Katheterablation konnte bislang nur für ausgewählte Patientengruppen nachgewiesen werden [10, 11]. Ein genereller prognoseverbessernder Effekt bei allen Patienten mit Vorhofflimmern ist derzeit nicht belegt und erfordert ein differenziertes individuelles Abwägen. Unabhängig davon wird zunehmend empfohlen, eine Katheterablation früh im Krankheitsverlauf zu erwägen, um die Progression zu persistierendem AF und die damit oftmals verbundenen schlechteren Ablationsergebnisse zu vermeiden [12].

Bei paroxysmalem AF kann die Katheterablation auf Basis aktueller Studiendaten bereits als Erstlinientherapie ohne vorherige antiarrhythmische medikamentöse Therapie zur Symptomverbesserung und Reduktion des Krankheitsprogresses erfolgen. Es sollte allerdings berücksichtigt werden, dass die Patienten in den zugrundeliegenden Studien oftmals unzureichend medikamentös antiarrhythmisch behandelt wurden [13]. Spätestens bei unzureichendem Therapieerfolg oder Unverträglichkeit medikamentöser Strategien ist die Ablation eindeutig indiziert [8].

**Fig. 1:**
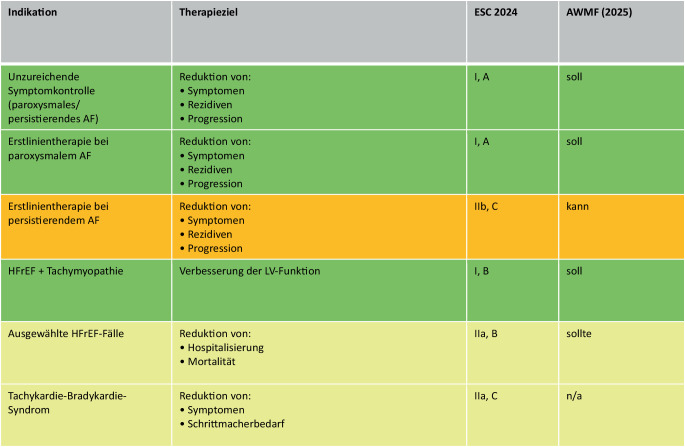
Leitlinienempfehlungen zur Indikationsstellung zur Therapie von Vorhofflimmern

Eine besonders klare Indikationsstellung besteht bei Patienten mit Herzinsuffizienz und reduzierter linksventrikulärer Ejektionsfraktion, insbesondere bei Verdacht auf eine AF-bedingte Tachymyopathie. In dieser Gruppe konnte gezeigt werden, dass die Ablation nicht nur die Symptomatik verbessert, sondern auch mit einer Reduktion herzinsuffizienzbedingter Hospitalisierungen und einer Senkung der Mortalität assoziiert ist (Fig. 1) [10, 11]. Entsprechend wird die Ablation hier mit hoher Evidenz empfohlen. Auch bei Patientinnen und Patienten mit Tachykardie-Bradykardie-Syndrom kann sie zur Symptomkontrolle beitragen und individuell eine Schrittmacherimplantation vermeiden.

### Fazit

Die Kenntnis der aktuellen Indikationen zur Katheterablation sowie eine sorgfältige individuelle Patientenevaluation sind entscheidend für eine adäquate Therapiestratifizierung. Die Indikationsstellung sollte stets im Rahmen einer partizipativen Entscheidungsfindung gemeinsam mit dem Patienten erfolgen.

## Pulmonalvenenisolation

Die interventionelle AF-Therapie basiert auf der elektrischen Isolation der Pulmonalvenen (PVI). Hierfür stehen thermische Verfahren wie Radiofrequenz- (RF) und Kryoablation sowie die Pulsed-Field-Ablation (PFA) als nichtthermisches Verfahren zur Verfügung. Während RF- und Kryoballonablation über viele Jahre als Goldstandard etabliert waren, gewinnt die PFA rasant an Bedeutung [14–16].

Bei der RF-Ablation gilt die zirkumferenzielle Isolation der ipsilateralen Pulmonalvenen mittels *Point-by-point-Technik* unter Nutzung dreidimensionaler Mapping-Systeme und gespülter Katheter als Standard [17, 18]. Zur Vermeidung von Pulmonalvenenstenosen wird eine antrale Ablationsstrategie empfohlen, die zugleich eine gewisse Substratmodifikation beinhaltet [19]. Der Einsatz von Anpressdruck-Kathetern zeigt zwar keinen konsistenten Sicherheitsvorteil, ist jedoch mit kürzeren Prozedurzeiten, höheren Raten einer sofortigen Pulmonalvenenisolation und höheren 1‑Jahres-Erfolgsraten assoziiert, insbesondere in Kombination mit Ablationsindizes wie „ablation index“ oder „lesion size index“ [20, 21]. Zunehmend wird auch die *High-power-short-duration(HPSD)-Ablation* eingesetzt, die bei vergleichbarem Sicherheitsprofil kürzere Applikationszeiten, geringere Rekonnektionsraten und höhere Rezidivfreiheit ermöglicht [22]. Neue Katheterdesigns könnten künftig zusätzliche Vorteile bieten.

Die Kryoballonablation ist als standardisiertes *Single-shot-Verfahren* etabliert und erlaubt die Isolation einzelner Pulmonalvenen mit einer Applikation. Die Katheterführung erfolgt überwiegend fluoroskopisch, wodurch die prozedurale Komplexität reduziert wird, allerdings bei tendenziell längerer Durchleuchtungszeit im Vergleich zur RF-Ablation. In randomisierten Studien wie FIRE AND ICE und CIRCA-DOSE erwies sich die Kryoballonablation der RF-Ablation hinsichtlich Effektivität und Sicherheit als nicht unterlegen [23]. Darüber hinaus konnte in Studien wie EARLY-AF auch eine Überlegenheit gegenüber antiarrhythmischer Medikation als Erstlinientherapie bei paroxysmalem Vorhofflimmern gezeigt werden. Auch bei Patienten mit kardialen Komorbiditäten, insbesondere Herzinsuffizienz, erwies sich das Verfahren als effektiv und sicher [24].

Die Pulsed-Field-Ablation ist ein nichtthermisches Verfahren, bei dem durch elektrische Impulse eine selektive Schädigung von Kardiomyozyten erreicht wird. Im Gegensatz zu thermischen Verfahren schont die PFA extrakardiale Strukturen weitgehend. Im multizentrischen MANIFEST-Register mit über 17.000 Prozeduren wurden keine atrioösophagealen Fisteln oder irreversiblen Phrenikusparesen beobachtet [16]. In der ADVENT-Studie zeigte sich PFA im 1‑Jahres-Follow-up hinsichtlich Effektivität als nicht unterlegen gegenüber thermischen Verfahren, bei vergleichbar niedriger Komplikationsrate. Auch in der SINGLE-SHOT-CHAMPION-Studie konnte die Nichtunterlegenheit gegenüber der Kryoballonablation bestätigt werden [25].

Ein wesentlicher Vorteil der PFA liegt in der vergleichsweise einfachen Handhabung und kurzen Prozedurzeiten [26]. Allerdings sind klassische elektrophysiologische Endpunkte wie Entrance- oder Exitblock durch reversible Effekte („stunning“) nur eingeschränkt beurteilbar. Für eine nachhaltige Läsionsbildung bleiben daher eine optimale Katheterpositionierung und ein möglichst optimaler Gewebekontakt entscheidend [27]. Direkte Vergleichsdaten zwischen verschiedenen PFA-Systemen sind bislang begrenzt; Langzeitergebnisse und potenzielle spezifische Komplikationen wie Hämolyse oder Koronarspasmen müssen weiter untersucht werden. Trotz der scheinbar geringeren prozeduralen Komplexität setzt auch die PFA ausreichende Erfahrung in linksatrialen Ablationsverfahren voraus.

### Fazit

Eine Pulmonalvenenisolation bleibt der zentrale Baustein zur Therapie von AF und wird derzeit vor allem mittels RF-, Kryoballon- und PFA-Technologie durchgeführt. Die Verfahren zeigen eine vergleichbare langfristige Effektivität. Moderne RF-Techniken mit Anpressdruckmessung, Ablationsindizes und HPSD können Prozedurdauer und Ergebnis optimieren. Die Kryoballonablation ist als standardisiertes Verfahren mit günstigem Sicherheitsprofil etabliert, während PFA als innovative Technologie mit potenziellen Vorteilen hinsichtlich Sicherheit und Effizienz gilt, deren Langzeitergebnisse weiter evaluiert werden müssen.

## Strategie für persistierendes Vorhofflimmern

Trotz erheblicher technologischer Fortschritte bestehen weiterhin nur wenige evidenzbasierte Empfehlungen für die Ablation des persistierenden AF über die Pulmonalvenenisolation hinaus. Eine zentrale Empfehlung besteht darin, bei allen Patienten die vollständige elektrische Isolation der Pulmonalvenen als primären Endpunkt sicherzustellen. Werden zusätzliche lineare Läsionen angelegt, sollte ein bidirektionaler Leitungsblock obligat nachgewiesen werden.

Für die Alkoholablation der V. Marshall besteht gemäß dem EHRA-Konsensuspapier eine optionale Indikation. Gleichzeitig wird ausdrücklich von einer substratbasierten Ablation auf Grundlage von MRT-Daten abgeraten. Weitere Strategien werden zwar diskutiert, sind jedoch aufgrund der limitierten Evidenz nicht empfohlen. Entsprechend sprechen sich die ESC-Leitlinien nicht für zusätzliche Strategien über die PVI hinaus aus, während in US-amerikanischen Leitlinien ein selektives Erwägen möglich ist.

Die Hinterwand des linken Vorhofs weist embryologische Gemeinsamkeiten mit den Pulmonalvenen auf und besitzt potenziell arrhythmogene Eigenschaften. Daraus ergibt sich die theoretische Überlegung, durch eine zusätzliche Isolation der linksatrialen Hinterwand weitere Trigger zu eliminieren [28]. Bisherige Studien konnten jedoch keinen konsistenten Vorteil dieser Strategie gegenüber einer alleinigen PVI zeigen [28].

Auch additive lineare Ablationsstrategien werden kontrovers diskutiert. Während solche Linien bei linksatrialen Tachykardien sinnvoll sein können, ist die Evidenz für einen präventiven Einsatz zum Vermeiden von AF-Rezidiven uneinheitlich. Neuere Studien deuten darauf hin, dass bei ausgewählten Patienten mit persistierendem AF und nachweisbarem Niedervoltage-Areal zusätzliche substratbasierte Strategien die Rezidivrate reduzieren können [29]. Demgegenüber konnten frühere randomisierte Studien wie STAR-AF II und CHASE-AF keinen Vorteil einer empirischen Substratmodifikation gegenüber einer alleinigen PVI zeigen [30, 31].

Die elektrische Isolation des linken Vorhofohrs stellt eine weitere potenzielle Strategie dar. In der BELIEF-Studie konnte bei Patienten mit langanhaltend persistierendem AF eine verbesserte Rezidivfreiheit gezeigt werden [32]. Die jüngere kontrollierte und randomisierte ASTRO-AF-Studie fand dagegen keinen Vorteil einer LAA-Isolation mittels Kryoballon gegenüber anderen Strategien bei Patienten mit bereits isolierten Pulmonalvenen. Insgesamt bleibt der Stellenwert dieser Technik unklar und sollte derzeit auf selektierte Patienten mit nachgewiesenen Triggern oder atrialen Tachykardien aus dem LAA beschränkt bleiben. Zudem wurde abhängig von der technischen Umsetzung eine erhöhte Rate thromboembolischer Ereignisse trotz adäquater Antikoagulation beschrieben [33].

Bei Rezidiven nach initial erfolgreicher Ablation empfehlen die ESC-Leitlinien eine erneute Ablation, sofern ein klinischer Nutzen bestand. Für die Wiederholungsprozedur wird empfohlen, nicht die initial verwendete Technologie erneut einzusetzen, sondern bevorzugt eine elektroanatomisch gesteuerte Punkt-für-Punkt-Ablation mittels Hochfrequenzstrom durchzuführen, insbesondere bei zusätzlich dokumentierten atrialen Tachykardien.

### Fazit

Die vollständige elektrische Isolation der Pulmonalvenen bleibt der zentrale Endpunkt der Ablation bei persistierendem Vorhofflimmern und sollte bei allen Patienten angestrebt werden. Werden zusätzliche lineare Läsionen angelegt, ist ein bidirektionaler Leitungsblock obligat.

## Komplikationen der Vorhofflimmerablation

Die Katheterablation des Vorhofflimmerns ist trotz technischer Fortschritte weiterhin mit relevanten Komplikationsrisiken verbunden. Die Kenntnis und das kompetente Management dieser Komplikationen ist ein zentraler Bestandteil der Qualitätssicherung und Voraussetzung für die Zertifizierung von Ablationszentren. Daher müssen standardisierte SOP für das frühzeitige Erkennen und das strukturierte Management von Komplikationen vorliegen. Diese sollten praxisnah sein und lokale Gegebenheiten wie Notfallkontakte, Verfügbarkeit von Blutprodukten sowie die Zusammenarbeit mit Neurologie und Herzchirurgie regeln.

Zu den klinisch relevantesten Komplikationen zählen Perikardtamponade, thromboembolische Ereignisse und – selten – atrioösophageale Fisteln. Diese sind potenziell lebensbedrohlich und erfordern eine sofortige Diagnostik und Therapie. Gefäßkomplikationen an den Leistenzugängen sind die häufigste Komplikationsgruppe und umfassen Hämatome, Pseudoaneurysmen oder arteriovenöse Fisteln. Viele Verläufe sind konservativ behandelbar, komplexere Fälle können jedoch interventionelle oder chirurgische Maßnahmen notwendig machen. Der Einsatz ultraschallgesteuerter Punktionen kann das Risiko senken [34].

Die Gesamtkomplikationsrate liegt konsistent zwischen 2,5 und 8 %, die Krankenhausmortalität in erfahrenen Zentren bei etwa 0,05–0,1 % [35]. Mit zunehmender Erfahrung sinkt die Komplikationsrate, was die Bedeutung strukturierter Ablationsprogramme und ausreichender Fallzahlen unterstreicht. Frauen weisen zudem ein erhöhtes Risiko für Komplikationen und längere Krankenhausaufenthalte auf [36].

Technologiespezifisch treten persistierende Phrenikusparesen überwiegend nach Kryoablation auf, während ösophageale Verletzungen vor allem bei RF-Ablation beschrieben sind. Für die PFA deuten erste Daten auf eine vergleichbare Gesamtkomplikationsrate bei geringerer Schädigung extrakardialer Strukturen hin. Beobachtet wurden jedoch spezifische Nebenwirkungen wie Hämolyse oder Koronarspasmen [37, 38].

Thromboembolische Ereignisse, insbesondere Schlaganfälle oder transitorische ischämische Attacken, treten in etwa 0,15–0,5 % der Fälle auf und manifestieren sich meist innerhalb der ersten 24 h, mit erhöhter Gefährdung in den ersten 2 Wochen nach Ablation [39]. Eine konsequente Antikoagulation mit ACT-Werten über 300 s sowie ein strukturiertes intraprozedurales Management sind entscheidend.

Die Perikardtamponade ist die häufigste potenziell lebensbedrohliche Komplikation und resultiert meist aus mechanischen Verletzungen oder thermischen Effekten während der Ablation. Klinisch zeigt sie sich durch Blutdruckabfall und hämodynamische Instabilität. Die rasche Diagnose mittels Echokardiographie und die sofortige Drainage sind essenziell. Für schwere Verläufe muss insbesondere in Zentren ohne eigene Herzchirurgie ein definiertes Vorgehen zur herzchirurgischen Versorgung gewährleistet sein.

Die Phrenikusparese tritt überwiegend bei Kryoablation auf und betrifft den rechten Nervus phrenicus. Eine kontinuierliche intraprozedurale Überwachung ist daher obligat. Bei suffizientem Monitoring der Phrenikusaktivität mit rechtzeitigem Abbruch der Kryoapplikation bildet sich die Parese spontan zurück und persistiert selten langfristig.

Pulmonalvenenstenosen sind aufgrund antraler Ablationsstrategien heute selten, können aber verzögert symptomatisch werden und erfordern dann eine gezielte Bildgebung. Weitere seltene, aber relevante Komplikationen sind Luftembolien und Koronararterienverletzungen, Letztere insbesondere bei bestimmten Ablationsarealen oder im Zusammenhang mit PFA.

### Fazit

Insgesamt ist die Katheterablation heute ein sicheres Verfahren, erfordert jedoch hohe prozedurale Expertise und strukturierte organisatorische Abläufe. Zentren mit höheren Fallzahlen weisen tendenziell niedrigere Komplikationsraten auf. Die umfassende Kenntnis potenzieller Komplikationen sowie klar definierte SOP sind essenziell für die Qualitätssicherung von Ablationszentren. Sie müssen praxisnah gestaltet und für alle Beteiligten jederzeit verfügbar sein, um im Komplikationsfall ein schnelles und strukturiertes Vorgehen zu ermöglichen.

## Ausblick und neue Entwicklungen

Die Katheterablation von AF hat sich seit der letzten Version dieses Positionspapiers im Jahr 2017 weiterentwickelt. Dies betrifft sowohl veränderte Empfehlungsgrade für die Indikation als auch neue Ablationsmodalitäten und innovative Strategien. Es besteht Konsens, dass selektionierte Patientengruppen hinsichtlich Symptom- und teilweise auch Prognoseverbesserung von einer Ablation profitieren. Weitere randomisierte Studien sind jedoch erforderlich, um die Rolle der Ablation als Erstlinientherapie bei Patienten mit Herzinsuffizienz und erhaltener linksventrikulärer Funktion abschließend zu klären. Diese Fragestellung wird derzeit in mehreren multizentrischen Studien untersucht [40].

Aktuell wird nach AF-Ablation eine orale Antikoagulation für mindestens 2–3 Monate empfohlen. Die weitere Fortführung richtet sich nach dem individuellen CHA_2_DS_2_-VA-Score und erfolgt unabhängig vom Ablationserfolg. Im klinischen Alltag wird diese Empfehlung jedoch häufig nicht konsequent umgesetzt. Aktuelle Studien zeigen, dass bei Patienten mit niedrigem thromboembolischen Risiko ein Jahr nach erfolgreicher PVI und damit einhergehender Freiheit von Vorhofflimmern auf eine weitere Antikoagulation verzichtet werden kann [41].

Eine alternative Strategie stellt die interventionelle Schlaganfallprophylaxe mittels Vorhofohrokkluder dar. In der OPTION-Studie wurde dieses Verfahren mit einer fortgesetzten DOAK-Therapie verglichen. Dabei zeigte sich über 3 Jahre eine signifikante Reduktion von Blutungsereignissen nach Vorhofohrverschluss. Für den kombinierten Endpunkt aus Mortalität, Schlaganfall und systemischer Embolie ließ sich bei limitierter statistischer Power jedoch keine abschließende Aussage treffen [42].

Insbesondere bei nichtparoxysmalem AF werden häufig zusätzliche Ablationsstrategien zur PVI eingesetzt. Diese umfassen sowohl empirische als auch individualisierte Konzepte, die in der Regel auf Mapping-Informationen basieren, etwa Niedervoltage-Areale oder charakteristische lokale Elektrogramme. Zunehmend wird untersucht, inwieweit künstliche Intelligenz die Identifikation kritischer Arrhythmiesubstrate unterstützen kann. Erste Studien zeigen, dass KI-gestützte Strategien zur Identifikation spatiotemporaler Dispersionsmuster mit einer Reduktion von AF-Rezidiven assoziiert sein können, wenngleich eine erhöhte Rate atrialer Tachykardien beobachtet wurde [43]. Perspektivisch könnte KI auch die Patientenselektion, die Wahl der optimalen Ablationsstrategie und das Management – etwa hinsichtlich der Antikoagulation – im Sinne einer individualisierten Therapie verbessern.

## Zertifizierung von Vorhofflimmerzentren der DGK

Katheterablationen zur Behandlung von AF sollten ausschließlich in qualifizierten und zertifizierten Zentren durch erfahrene Operateure durchgeführt werden. Die Zertifizierung durch die DGK bewertet neben der Indikation und Therapie auch strukturelle, organisatorische und infrastrukturelle Voraussetzungen. Ein wesentlicher Bestandteil ist die Sicherstellung einer hohen Prozessqualität, insbesondere im Management potenzieller Komplikationen.

Ziel der Zertifizierung ist die Etablierung und Dokumentation einer standardisierten, qualitätsgesicherten Versorgung von Patientinnen und Patienten mit Vorhofflimmern einschließlich der Katheterablation. Grundlage bilden definierte Kriterien der DGK, deren Einhaltung im Rahmen eines Peer-Review-Verfahrens überprüft wird (Tab. 1).

**Tab. 1:** Kriterien für die Zertifizierung als Vorhofflimmerzentrum

Kriterium	Aktuell	Vorher	Anmerkungen
Mindestanzahl jährliche Vorhofflimmerablationen/Zentrum	150	75	–
Rezertifizierung	Nach 3 Jahren	Erstmalig nach 3 Jahren, dann alle 5 Jahre	Anpassung an die IQTIG-Qualitätskriterien
Rhythmologische Struktur zur tagesgleichen Entlassung	Keine eigenständige Rhythmusambulanz gefordert, jedoch standardisierte Nachsorge (ggf. mit niedergelassenen Kardiologen)	Eigenständige Rhythmusambulanz zur Nachsorge gefordert	–
Präinterventionelle Indikationsstellung	Struktur erforderlich (z. B. prästationäre Visite) mit umfassender mündlicher und schriftlicher Aufklärung vor dem Eingriff	Keine Vorgabe	–
Analgosedierung	Ärztliches und Assistenzpersonal muss ausreichend im Bereich Sedierung geschult sein	Keine Vorgabe	–
Nachsorge/Überwachung	Individuell festgelegte Überwachungsdauer, ggf. < 24 h; telemetrische Überwachung abhängig von Patientensituation	Überwachung 24–48 h; mindestens 12 h EKG-Telemetrie	–

## Data Availability

Für diesen Beitrag wurden keine Studien durchgeführt.

## Anhang

**Tab. 3:** Angegebene Interessenskonflikte der Autoren.

	Autoren										
	CProf. Dr. Daniel Steven	CProf. Dr. K. R. Julian Chun	Prof. Dr. Isabel Deisenhofer	Prof. Dr. Thomas Deneke	CPriv.-Doz. Dr. Maria Papathanasiou	Prof. Dr. Andreas Rillig	CProf. Dr. Boris Schmidt	Prof. Dr. Christian Veltmann	Prof. Dr. Stephan Willems	Prof. Dr. Maura Magdalena Zylla	Prof. Dr. Lars Eckardt
Berater- bzw. Gutachtertätigkeit oder bezahlte Mitarbeit in einem wissenschaftlichen Beirat eines Unternehmens der Gesundheitswirtschaft (z. B. Arzneimittelindustrie, Medizinproduktindustrie), eines kommerziell orientierten Auftragsinstituts oder einer Versicherung	Abbott 3000 €, Biosense 2000 €, Boston Scientific 1500 €	Medtronic: Berater Boston Scientific: Berater	–	Nein	Beratertätigkeit für folgende Unternehmen: NovoNordisk, Cytokinetics, Bristol Myers Squibb, Bayer, Alnylam	Medtronic, Atricure, Johnson&Johnson, Boston Scientific	Boston Scientific, J&J Medtech und Abbott	Medtronic, Biotronik	Consultingvertrag mit Boston Scientific	Nein	Nein
Honorare für Vortrags- und Schulungstätigkeiten oder bezahlte Autoren- oder Co-Autorenschaften im Auftrag eines Unternehmens der Gesundheitswirtschaft, eines kommerziell orientierten Auftragsinstituts oder einer Versicherung	Abbott 2000 €	Medtronic: Vorträge, Schulungen Boston Scientific: Vorträge, Schulungen Abbott: Vorträge J&J Medtech: Vorträge	–	Biotronik, Abbott	Vortragstätigkeit für folgende Unternehmen: AstraZeneca, Alnylam, Bayer, Cytokinetics, Pfizer, Janssen, ZOLL	Medtronic, Atricure, Boston Scientific, LifeTech, Boehringer Ingelheim, Radcliffe Cardiology, Galvanize, Novartis, Bayer, Vector Medical	Boston Scientific und Abbott	Medtronic, BMS, Zoll	Referentenverträge mit folgenden Firmen: Abbott, BMS, Boston Scientific, Biotronik, Medtronic, Autorenschaft: Thieme Verlag	Astra Zeneca, Abbott, Boston Scientific	Verschiedene Vortragstätigkeiten für pharmazeutische Unternehmen und medizinische Firmen
Finanzielle Zuwendungen (Drittmittel) für Forschungsvorhaben oder direkte Finanzierung von Mitarbeitern der Einrichtung von Seiten eines Unternehmens der Gesundheitswirtschaft, eines kommerziell orientierten Auftragsinstituts oder einer Versicherung	Abbott 10.000 €	Boston Scientific	–	Nein	Nein	Medtronic, Else Kröner Fresenius	Boston Scientific, Abbott, Medtronic	Nein	Research grant Abbott und Boston Scientific	Bayer, Boston Scientific, DZHK	Nein
Eigentümerinteresse an Arzneimitteln/Medizinprodukten (z. B. Patent, Urheberrecht, Verkaufslizenz)	Nein	Nein	–	Nein	Nein	Nein	Nein	Nein	Nein	Nein	Nein
Besitz von Geschäftsanteilen, Aktien, Fonds mit Beteiligung von Unternehmen der Gesundheitswirtschaft	Bayer, Biontec	Nein	–	Nein	Nein	Nein	Nein	Nein	Nein	Nein	Nein
Persönliche Beziehungen zu einem Vertretungsberechtigten eines Unternehmens Gesundheitswirtschaft	Nein	Nein	–	Nein	Nein	Nein	Nein	Nein	Nein	Nein	Nein
Mitglied von in Zusammenhang mit der Leitlinienentwicklung relevanten Fachgesellschaften/Berufsverbänden, Mandatsträger im Rahmen der Leitlinienentwicklung	S3 AWMF Leitlinie	EHRA SIC: Chair 2022–2024 EHRA SIC: Chair: 2024–2026	–	Heart Rhythm Society, European Heart Rhythm Association	Nein	AGEP, EHRA, AFNET	Nein	DGK, ESC, EHRA	Deutsche Gesellschaft für Kardiologie	Deutsche Gesellschaft für Kardiologie (Nukleusmitglied AG 28; Mitglied AGEP, AG 10, Sektion Young DGK, Basic Science Cluster) European Society of Cardiology (ESC) European Heart Rhythm Association (EHRA) (Mitglied Scientific Initiatives Committee, Mitglied AQIHEC)	DGK, EHRA, ESC
Politische, akademische (z. B. Zugehörigkeit zu bestimmten „Schulen“), wissenschaftliche oder persönliche Interessen, die mögliche Konflikte begründen könnten	Nein	Nein	–	Nein	Nein	Nein	Nein	Nein	Nein	Nein	Nein
Gegenwärtiger Arbeitgeber, relevante frühere Arbeitgeber der letzten 3 Jahre	Uniklinik Köln AöR	CCB Frankfurt am Main	–	Klinikum Nürnberg, Herz- und Gefäßklinikum Campus Bad Neustadt	Universitätsklinikum Frankfurt	Universitätsklinikum Hamburg-Eppendorf	MVZ CCB Frankfurt und Main-Taunus GbR, Agaplesion Markus Krankenhaus	Selbstständig	Asklepios Klinikum St. Georg, Hamburg	Universitätsklinikum Heidelberg	Univeritätsklinikum Münster Klinik für Kardiologie II: Rhythmologie
